# Abnormal mannose-6-phosphate receptor trafficking impairs recombinant alpha-glucosidase uptake in Pompe disease fibroblasts

**DOI:** 10.1186/1755-8417-1-6

**Published:** 2008-12-01

**Authors:** Monica Cardone, Caterina Porto, Antonietta Tarallo, Mariella Vicinanza, Barbara Rossi, Elena Polishchuk, Francesca Donaudy, Generoso Andria, Maria Antonietta De Matteis, Giancarlo Parenti

**Affiliations:** 1 Telethon Institute of Genetics and Medicine, Via Castellino, 80131 Naples, Italy; 2 Department of Paediatrics, Federico II University, Via S. Pansini, 80131 Naples, Italy; 3 Department of Cell Biology and Oncology, Consorzio Mario Negri Sud, 66030 Santa Maria Imbaro, Chieti, Italy

## Abstract

**Background:**

Pompe disease (PD) is a metabolic myopathy caused by α-glucosidase (GAA) deficiency and characterized by generalized glycogen storage. Heterogeneous *GAA *gene mutations result in wide phenotypic variability, ranging from the severe classic infantile presentation to the milder intermediate and late-onset forms. Enzyme replacement therapy (ERT) with recombinant human GAA (rhGAA), the only treatment available for PD, intriguingly shows variable efficacy in different PD patients. To investigate the mechanisms underlying the variable response to ERT, we studied cell morphology of PD fibroblasts, the distribution and trafficking of the cation-independent mannose-6-phosphate receptor (CI-MPR) that mediates rhGAA uptake, and rhGAA uptake itself.

**Results:**

We observed abnormalities of cell morphology in PD cells. Electron microscopy analysis showed accumulation of multivesicular bodies and expansion of the Golgi apparatus, and immunolocalization and western blot analysis of LC3 showed activation of autophagy. Immunofluorescence analysis showed abnormal intracellular distribution of CI-MPR in PD fibroblasts, increased co-localization with LC3 and reduced availability of the receptor at the plasma membrane. The recycling of CI-MPR from the plasma membrane to the trans-Golgi network was also impaired. All these abnormalities were more prominent in severe and intermediate PD fibroblasts, correlating with disease severity. In severe and intermediate PD cells rhGAA uptake and processing were less efficient and correction of GAA activity was reduced.

**Conclusion:**

These results indicate a role for disrupted CI-MPR trafficking in the variable response to ERT in PD and have implications for ERT efficacy and optimization of treatment protocols.

## Background

Pompe disease (PD, glycogenosis type II) is a metabolic myopathy, with an estimated incidence of 1:40,000, characterized by intra-lysosomal glycogen storage [1]. PD is caused by mutations in the acid α-glucosidase (*GAA*) gene, encoding the lysosomal hydrolase α-glucosidase (acid maltase, GAA, E.C.3.2.1.20). GAA is synthesized in the endoplasmic reticulum as a 110 kDa precursor, which undergoes N-glycan processing in the Golgi apparatus, and is proteolytically processed in the lysosomes into active polypeptides of 76 and 70 kDa, through an intermediate molecular form of 95 kDa [2,3]. In PD, glycogen accumulation occurs in almost every organ and system, but is particularly evident in skeletal muscle and heart, the sites of the most debilitating clinical manifestations [1]. Different mutations of the *GAA *gene result in a wide phenotypic spectrum, with respect to age of onset of manifestations, rate of disease progression and variable association of symptoms. The different clinical forms of PD range from a devastating classic infantile phenotype, characterized by early onset, severe cardiomyopathy and early lethality, to intermediate phenotypes and late onset (childhood, juvenile or adult) forms in which cardiac involvement is absent or mild [1,4,5].

The past two decades have been characterized by impressive progress in the treatment of lysosomal storage diseases (LSDs), with the development of innovative therapies, including haematopoietic stem cell transplantation (HSCT) [6], enzyme replacement therapy (ERT) [7], substrate reduction therapy (SRT) [8] and enzyme enhancement therapy (EET) by pharmacological chaperons [9,10]. Among these approaches, ERT represented a major breakthrough in the treatment of LSDs, initially used for the treatment of Gaucher disease and now available for several other LSDs.

ERT with recombinant human GAA (rhGAA) is presently the only approach for the treatment of PD patients. The first clinical trials, based on the use of an enzyme derived from transgenic rabbits, demonstrated that ERT was effective in improving cardiomyopathy, survival, growth and motor function in classic infantile PD patients [11]. ERT has been subsequently extended to PD patients with other phenotypes. The results of the published studies showed dramatic improvements of cardiac, respiratory and motor function in some patients, whereas in others ERT failed to cause significant clinical improvement [12,13]. This suggests that the clinical outcome in response to ERT may be variable, correlating with histological evidence of poor glycogen clearance [14], and that correction of glycogen storage in skeletal muscle is particularly challenging. Factors such as age at the start of treatment, stage of skeletal muscle damage, antibody responses [15], insufficient targeting of rhGAA to skeletal muscle and high clearance of the enzyme by the liver [16] play a role in ERT efficacy, although the reasons for the variable responses of different PD patients are not completely clear and other factors, such as patients' genotype and abnormalities of cell functions, may also be implicated.

We postulated that an impairment of house-keeping cellular functions and membrane trafficking resulting from abnormal substrate storage is an additional and important factor influencing the efficacy of ERT in PD, and possibly in other LSDs.

To date, several studies have pointed to the role of a variety of structural and biochemical responses triggered by intracellular storage, which are considered to be responsible for the pathogenetic manifestation of LSDs. These abnormalities include activation of inflammation by cytokines, impairment of the autophagic pathway, alterations of signal transduction pathways, altered calcium homeostasis and apoptosis and abnormalities of intracellular trafficking [17,18].

Abnormal intracellular trafficking of lipids and proteins may affect the function of membrane-bound proteins, such as receptors, and ligands. The cation-independent 300 kDa mannose-6-phosphate receptor (CI-MPR) is of particular interest as it is a key player in the internalization of exogenous enzymes, and for the possible consequences of deranged CI-MPR function on ERT efficacy. CI-MPR is an integral membrane glycoprotein that follows a complex and finely regulated itinerary from the trans-Golgi network (TGN), where it binds newly synthesized lysosomal hydrolases, travels through the early endosomes towards the late endosomal compartments, and recycles back to the TGN. At the steady state, a fraction (approximately 10%) of CI-MPR is located on cell surface, where it mediates the uptake of lysosomal enzymes, is internalized in the early endocytic compartment and then recycled again to the plasma membrane through the endocytic recycling compartment (ERC) [19].

To address the consequences of abnormalities of cellular morphology and function on CI-MPR subcellular localization, we studied fibroblasts from PD patients with different genotypes and phenotypes. We demonstrated that in these cells, which showed abnormalities of cellular morphology, CI-MPR is mislocalized and its availability at the plasma membrane is reduced. These abnormalities in CI-MPR distribution result in a less efficient uptake of rhGAA by PD fibroblasts.

## Results

### PD fibroblasts show abnormalities of cell morphology

In order to determine whether PD fibroblasts display abnormalities of cell morphology and trafficking we took advantage of five fibroblast cell lines from PD patients with different genotypes and associated phenotypes (Table 1). All cell lines studied had nearly absent or reduced GAA activity due to mutations in the *GAA *gene, compared with age-matched control fibroblasts.

**Table 1:** Pompe disease fibroblast cell lines, phenotype, genotype and residual GAA activity

**Cell line**	**Phenotype**	**Genotype**		**GAA relative activity**
			
		**DNA mutation**	**Protein mutation**	
1	severe	c.1101G>A/c.1927G>A	p.W367X/p.G643R	0.3%
2	intermediate	c.1655T>C/c.1655T>C	p.L552P/p.L552P	1.2%
3	intermediate	c.1655T>C/c.-35C>A	p.L552P/abn. splicing	1.6%
4	juvenile	c.1645G>C/c.692+1G>C	p.G549R/abn. splicing	3.2%
5	juvenile	c.-45T>G/unknown	abn. splicing/unknown	5.2%

GAA relative activity is expressed as percentage of normal (100%). Normal range of GAA activity in fibroblasts 58.5 +/- 28.1

**Figure 1:**
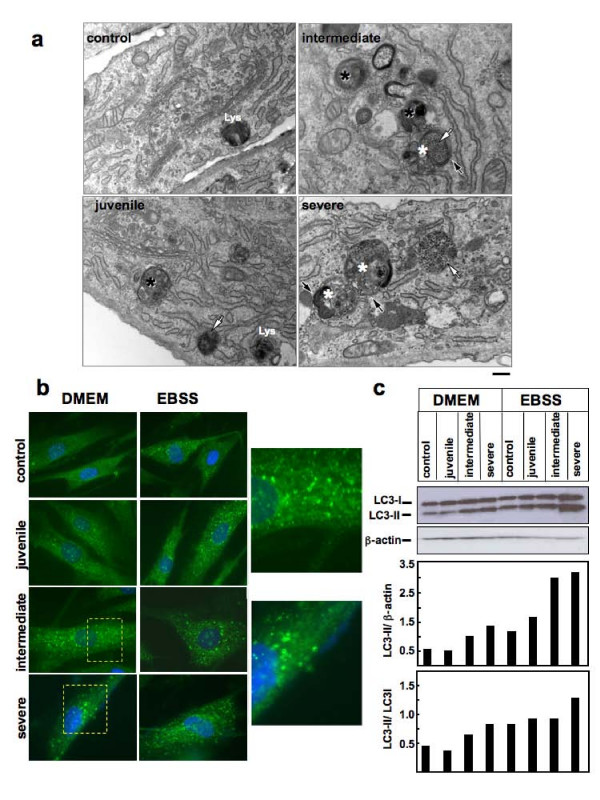
**Pompe disease fibroblasts show expansion of the autophagic compartment**. A) Electron microscopy of control and juvenile human fibroblasts (left panel) and intermediate and severe human fibroblasts (right panel). The latter two cell lines show increased number of multivesicular bodies (black asterisks) and of autophagosomes or autolysosomes (white asterisks) characterized by double membranes (black arrows) and cytoplasmic content. Also evident is the glycogen accumulation in Pompe disease (PD) cells (white arrows). Lysosomes are indicated. Bars 200 nm. (B) LC3 staining in control and PD fibroblasts grown in complete medium (left panel) or starved in amino acid-free medium for 2 hours (right panel). Magnification 63×. Cells were fixed, permeabilized and immunostained with a polyclonal antibody to endogenous LC3 and visualized by fluorescence microscopy. LC3 staining shows multiple vesicles in intermediate and severe PD patients consistent with autophagosomes. Insets show high magnification views of autophagosomes. (C) Western blot analysis of LC3 in control and PD fibroblasts. Lanes 1 to 4: cells grown in complete medium; lanes 5 to 8: cells starved in amino acid-free medium for 2 hours. Quantitative analysis by Image J of LC3-II peptides, normalized as ratio of LC3-II/β-actin (top) and LC3-II/LC3-I (bottom), showed that intermediate and severe PD fibroblasts have increased amounts of LC3-II as compared with control and juvenile PD fibroblasts.

Electron microscopy (EM) analysis of PD cells showed intralysosomal storage of glycogen, a typical hallmark of the disease [1,14,20], and an increased number of large membrane-bound structures containing intralumenal stacked, reticulated or whorled osmiophilic membranes known as 'myelin figures'. Some of these structures had features typical of endosomal multivesicular bodies (MVBs) (Figure 1a, black asterisks), while others exhibited features typical of autophagosomes (white asterisks), with double membranes (black arrows) and cytoplasmic-like content. In intermediate and severe PD fibroblasts, glycogen storage (white arrows) was evident as electron-dense spots either within endolysosomes and autophagosomes or dispersed in the cytoplasm. The percentage of endolysosome volume occupied by glycogen ranged from 14% in juvenile PD fibroblasts, to 43% in intermediate PD fibroblasts to 65% in severe PD fibroblasts. On average, MVBs and autophagosomes appeared to be more prominent in cells from severely affected patient as compared with control cells and with cells from patients with milder phenotypes, suggesting an expansion of the autophagic compartment in severe PD fibroblasts.

This finding was also confirmed by immunofluorescence and western blot analysis of the microtubule-associated protein 1 light chain 3 (LC3). LC3, encoded by the mammalian homolog of the yeast *atg8 *gene, and its processed phospholipid-conjugated LC3-II molecular form, localized in the autophagosomes, are known markers of autophagy [21-23]. Fibroblasts were studied both under standard culture conditions in DMEM medium and after starvation (amino acid deprivation for 2 hours), a stimulus for autophagy [22,23].

Immunofluorescence microscopy (Figure 1b) showed punctated LC3 signal in patients' cells either under basal conditions or after starvation. The LC3 signal was localized peripherally in multiple vesicles or cup-shaped structures, with a pattern compatible with a membrane-anchored protein localized in the autophagosomal compartment. This pattern was most prominent in PD fibroblasts from patients with the severe and intermediate forms. Starvation of cells in amino acid-deprived EBSS medium resulted in increased LC3 signal in controls and in juvenile PD fibroblasts, whereas no substantial change in LC3 staining was appreciable in severe and intermediate PD cells. To perform a quantitative analysis of LC3-positive spots at steady state and under basal culturing conditions, confocal images from control and PD fibroblasts stained with anti-LC3 antibody were processed by ImageJ (using the analysing particles module and considering as minimum size four pixels). The cells from controls and severe PD were classified into three groups based on the number of LC3-positive spots (1 to 10 spots, 11 to 40 spots and 41 to 200 spots). The analysis showed that the population of cells containing increased number of LC3-positive spots was more represented in severe PD fibroblasts (Table 2).

**Table 2:** LC3 positive spots in PD fibroblast cell lines

**Samples**	**Percentage of cells with 1 to 10 of LC3 spots**	**Percentage of cells with 11 to 40 of LC3 spots**	**Percentage of cells with 41 to 200 of LC3 spots**
Control	75%	20%	5%
Severe	10%	10%	80%

Western blot analysis (Figure 1c) of LC3 showed results consistent with those of immunofluorescence analysis in cells cultured under basal conditions, with an increased amount of LC3-II in cells from intermediate and severe PD patients compared with controls. After starvation all cells showed increased LC3-II expression. The ratio between LC3-II and LC3-I was increased in severe and intermediate PD fibroblasts under basal conditions, compared with controls and juvenile PD cells.

**Figure 2:**
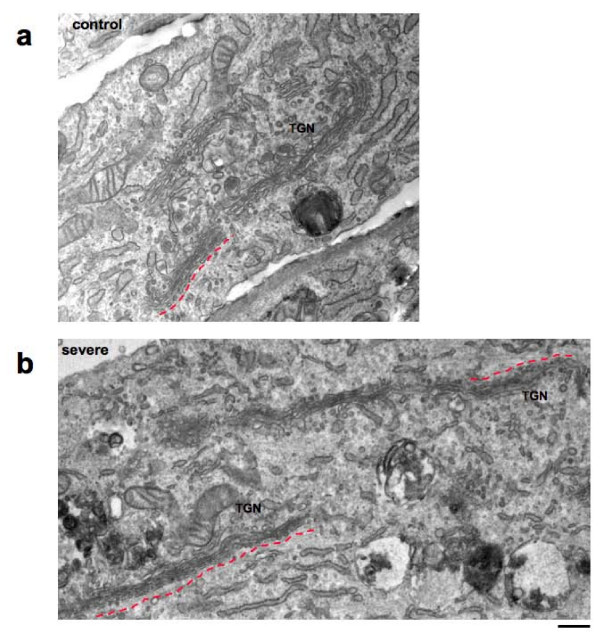
**Pompe disease fibroblasts show expanded Golgi apparatus**. Electron micrographs of severe Pompe disease fibroblasts (b) show longer Golgi cisternae. The cis-side (dotted red line) and trans-side (trans-Golgi network) of the Golgi apparatus are shown.

EM analysis showed that the overall organization of the Golgi stacks and the number of cisternae per stack (four cisternae/stack) were preserved in PD fibroblasts compared with control cells; however, the average length of Golgi cisternae, ranging from 600 nm to 1 μm in control fibroblasts, reached 2 μm in fibroblasts from patients with severe and intermediate PD (Figure 2b).

**Figure 3:**
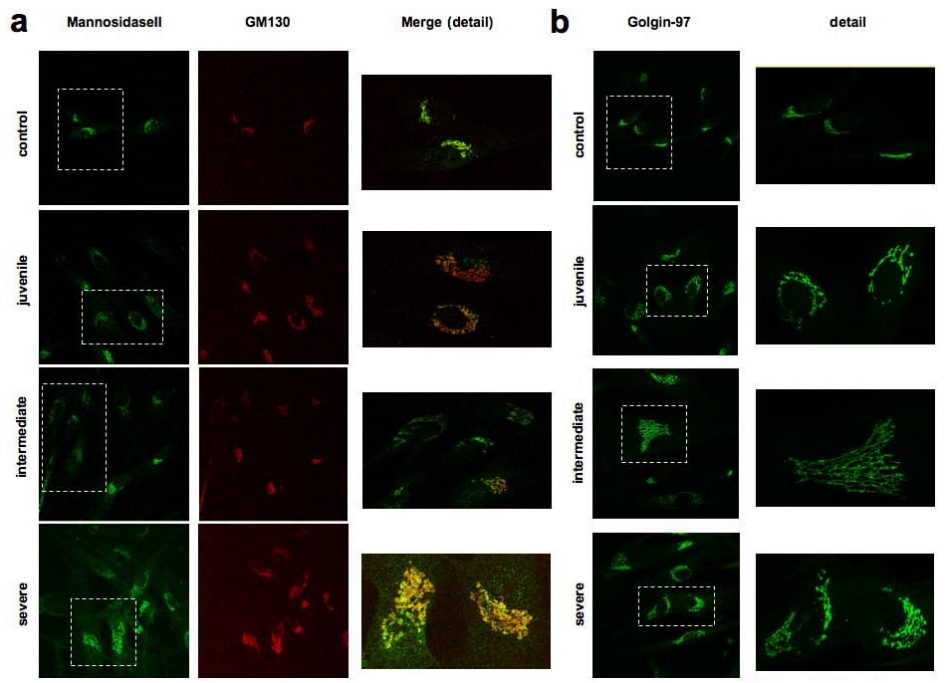
**Pompe disease fibroblasts show abnormal distribution of Golgi and trans-Golgi network markers**. Immunofluorescence analysis of markers of the Golgi apparatus. The cells were visualized by confocal fluorescence microscopy. Insets show high magnification views. The merged image of double staining is shown. The cells were fixed, permeabilized and stained with anti-Mannosidase II (green), anti-GM130 (red) (A), and with anti-Golgin 97 (green) (B). All markers showed abnormal distribution and expansion of the Golgi apparatus in intermediate and severe Pompe disease fibroblasts.

We also studied the distribution of several Golgi markers at immunofluorescence level, including that of GM130 and mannosidase II, markers of the cis and medial Golgi apparatus (Figure 3a), of golgin-97 (Figure 3b), γ-adaptin and TGN46 (not shown), which are localized in the TGN. The staining pattern of these Golgi markers consisted of a compact perinuclear structure in control cells and of a more reticular distended structure in PD fibroblasts (consistent with the increased cisternae length observed at EM). The 'distension' of the Golgi staining pattern was more prominent in severe and intermediate PD fibroblasts, thus apparently correlating with disease severity.

### CI-MPR is mislocalized and its availability at the plasma membrane is reduced in PD fibroblasts

The uptake of exogenous lysosomal enzymes at the plasma membrane and their delivery to lysosomes is performed by the CI-MPR [19]. Thus a correct CI-MPR localization and function is relevant for the efficacy of ERT.

**Figure 4:**
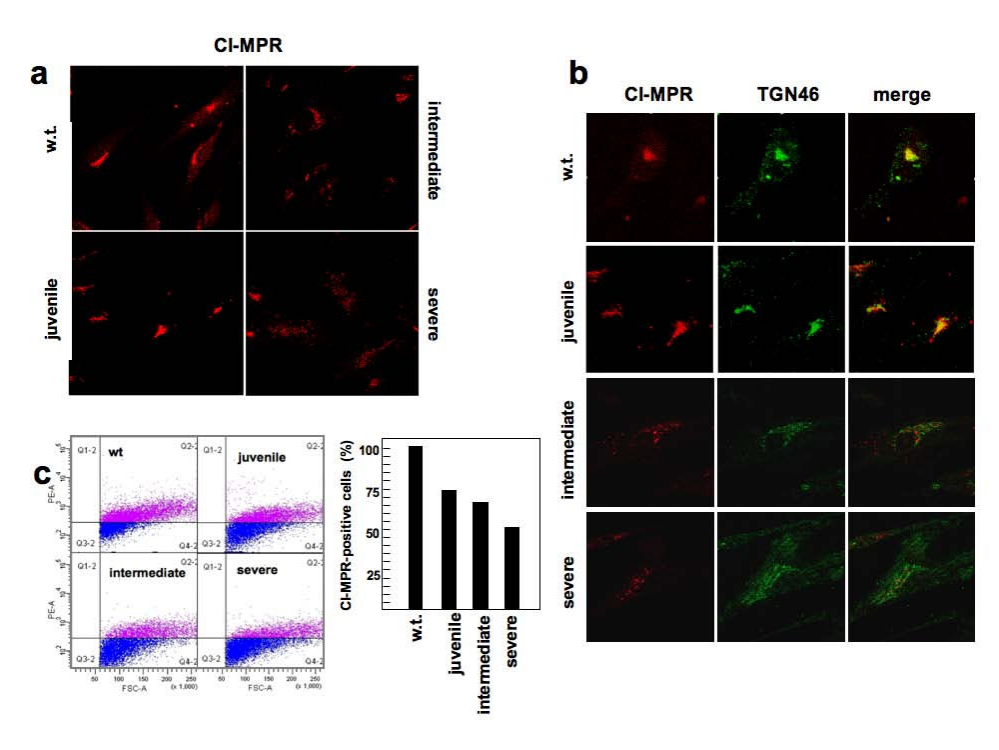
**Pompe disease fibroblasts show abnormal intracellular localization of CI-MPR and reduced availability at the plasma membrane**. (A) Immunofluorescence analysis of CI-MPR steady-state distribution in control and Pompe disease (PD) fibroblasts. The cells were grown on coverslips, fixed, permealized and stained with a mouse monoclonal antibody to CI-MPR. In intermediate and severe PD fibroblasts 55% and 75% of cells respectively showed reduced or mislocalized signal (right panel). Quantitative analysis was performed on 40 cells. (B) Co-staining of CI-MPR and TGN46. Cells were treated as above, stained with Alexa 594-conjugated donkey anti-mouse IgG (red channel) and Alexa 488-conjugated donkey anti-rabbit IgG (green channel) and visualized by confocal fluorescence microscopy. The right columns show the merged images of double staining of CI-MPR and TGN46. Intermediate and severe PD fibroblasts show abnormal distribution of both markers. (C) FACS analysis of CI-MPR expression at the plasma membrane of control and PD fibroblasts. Cells were incubated in ice with an anti-CI-MPR and a secondary antibody labelled with phycoerythrin (PE). The rate of cells expressing CI-MPR at the plasma membrane in PD fibroblasts is reduced in PD fibroblasts compared with controls and correlates with disease severity. The percentage is calculated by assuming that in control fibroblasts the rate is 100%. The data are representative of two independent experiments.

We studied CI-MPR distribution by confocal analysis of PD fibroblasts, and compared the results with those obtained from age-matched control fibroblasts. Scoring of CI-MPR distribution showed a high rate of cells with reduced or dispersed signal in severe PD (75% of the cells) and intermediate (55%) PD fibroblasts as compared with the compact juxtanuclear pattern observed in control cells (Figure 4a). Also the co-localization with the TGN marker TGN46 was poorer in intermediate and severe PD fibroblasts as compared with control cells (Figure 4b). Fibroblasts from patients with juvenile PD did not show substantial abnormalities of the distribution of CI-MPR or of TGN46.

We evaluated the amount of CI-MPR at the plasma membrane in PD and control fibroblasts as the availability of CI-MPR at the plasma membrane is of particular interest for its involvement in the uptake of exogenous enzymes and in the efficacy of ERT. To this end, cells were incubated with an anti-CI-MPR antibody in ice-cold medium and then analyzed on a fluorescence-activated cell sorter (FACS). Under these conditions, internalization of the CI-MPR-antibody complexes located on plasma membrane is prevented, and the anti-MPR antibody labels only the CI-MPR exposed at the plasma membrane. The percentage of CI-MPR positive cells was normalized, taking that observed in control fibroblasts as 100%. The number of CI-MPR-positive cells was significantly reduced in two (severe and intermediate) PD fibroblast cell lines (Figure 4c), with average values of 45 and 65%, respectively. In a juvenile PD cell line the percentage of positive cells was also reduced, albeit to a lesser extent (75%).

### The recycling of CI-MPR from the plasma membrane is reduced in PD fibroblasts

To investigate the trafficking of CI-MPR through the endocytic pathway, an essential route for the internalization of exogenous lysosomal enzymes, we applied a synchronization protocol including a pulse at 18°C with anti-CI-MPR antibody and then a chase at 37°C, in order to follow how much of the pool of plasma membrane receptor is able to reach early and late endosomes. Low temperature is permissive for transport from the plasma membrane to early endosomal compartment but delays the recycling pathway and the kinetics of endosome acidification [24].

**Figure 5:**
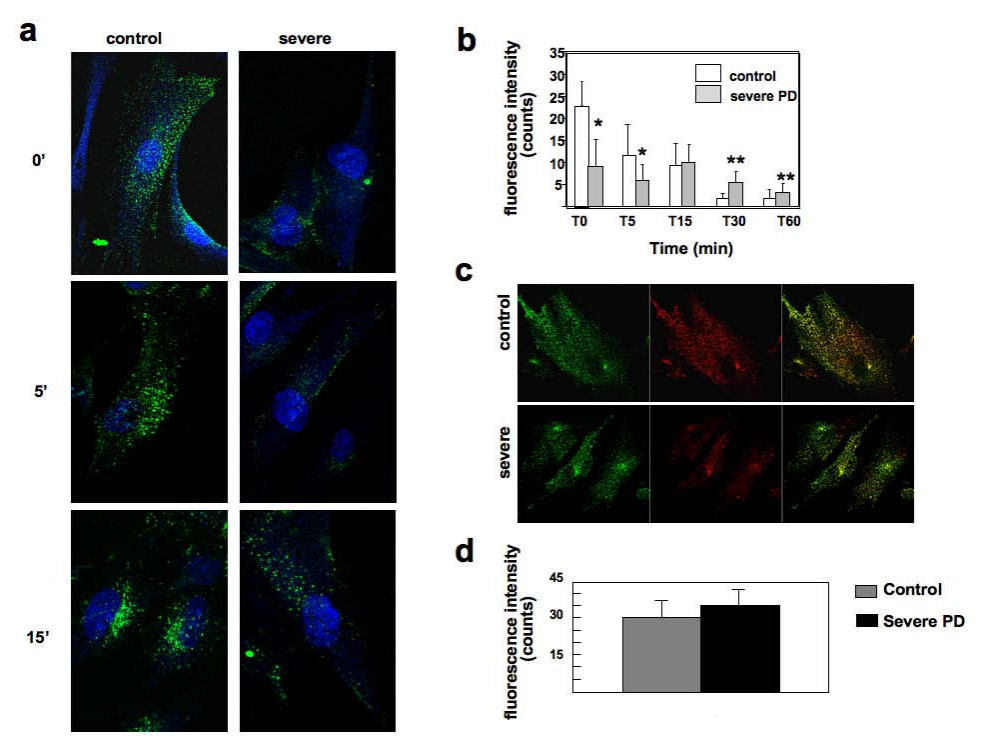
**Endocytic recycling of CI-MPR is impaired in Pompe disease fibroblasts**. To study the trafficking of CI-MPR through the endocytic pathway normal and severe fibroblasts grown on coverslip were incubated at 18° C for 1h with 15 ng/ml Alexa-488-anti-CI-MPR antibody. The cells were washed and fixed (T0) or warmed to 37°C for 5, 15, 30, 60 min (T5, T15, T30, T60) before being fixed, permeabilized and stained with Alexa 488-conjugated donkey anti-mouse IgG to amplify the signal.   (A) In control fibroblasts the CI-MPR signal reaches its steady-state within 15 minutes. In severe fibroblasts signal intensity is reduced and the correct localization is not reached. (B) Average fluorescence intensity of internalized anti-CI-MPR antibody was quantified within the area concerning each single cell, using LSM 3.2 software (Zeiss). The analysis was performed on 40 cells. The quantitative analysis of fluorescence intensity (time 0-60 min) shows progressive decrease of signal intensity in control fibroblasts. In severe Pompe disease (PD) fibroblasts the amount of fluorescence remains fairly constant, leading to an inversion of the rate between control and PD cells after 15 to 30 minutes. The error bars indicate the standard error. *=p≤0.05, **=p≤0.001. (C) The distribution of transferrin (Tf) and of trasferrin receptor (TR) is normal in PD fibroblasts. The cells were incubated with 50 mg/ml Alexa-488 transferrin at 18° C for 60 min, washed, fixed, permeabilized and stained with a trasferrin receptor antibody and Alexa-fluor 594 anti-rabbit IgG  antibody. Stained cells were visualized by confocal fluorescence microscopy. (D) Average fluorescence intensity of A488-Tf was quantified within the area concerning each single cell, using LSM 3.2 software (Zeiss). The analysis was performed on 40 cells. Quantitative analysis of fluorescence intensity of the Tf signal shows comparable results in control and severe PD fibroblasts (p≤0.026). The error bars indicate the standard error.

In control fibroblasts the CI-MPR signal was detectable in early endosomal structures during the 18°C block, and reached the juxtanuclear localization typical of the steady-state within 15 minutes after the temperature shift. In PD cells the CI-MPR signal intensity was reduced at 18°C and the CI-MPR was never observed to reach the perinuclear area after the temperature shift (Figure 5a).

The intensity of fluorescence was quantified, as described in Methods, up to 60 minutes after the temperature shift (Figure 5b). In control fibroblasts the intensity progressively decreased over time. This reduction was probably due to loss of fluorescent antibodies after several rounds of CI-MPR recycling and/or delivery of the anti-CI-MPR antibody to the late endosomes/lysosomes. In PD fibroblasts the initial amount of fluorescence measured at 18°C was reduced but the internalized fluorescence remained fairly constant, leading to an inversion of the rate between control and PD cells 15 to 30 minutes after the temperature shift (Figure 5b). This indicates that the antibody-CI-MPR complexes are sequestered in PD cells and do not efficiently recycle to the plasma membrane and/or deliver the antibody to the late endosomes/lysosomes.

To assess whether the recycling defect of CI-MPR observed in PD fibroblasts was accompanied by a general defect in clathrin-mediated endocytosis we analyzed the uptake of transferrin (Tf). The internalization of fluorescent Tf in the early endocytic compartment was studied, keeping the cells at 18°C for 60 minutes. The distribution of Tf and transferrin receptor (TR) in control and mutant cells was not substantially different (Figure 5c), suggesting that PD cells do not have a general defect in clathrin-mediated endocytosis but a selective impairment of CI-MPR trafficking and recycling along the endocytic pathway.

**Figure 6:**
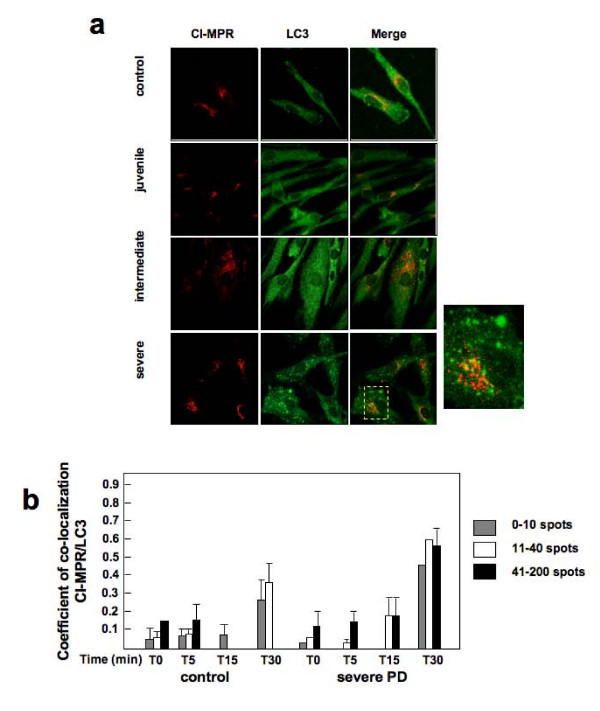
**CI-MPR co-localizes partially in LC3 positive vesicle in Pompe disease fibroblasts**. (A) Steady-state analysis of CI-MPR and LC3 positive vesicles shows co-localization in severe and intermediate Pompe disease (PD) fibroblasts. The cells were fixed, permeabilized and stained with a mouse monoclonal antibody to CI-MPR and a rabbit polyclonal antibody to LC3 and Alexa 594-conjugated donkey anti-mouse IgG (red channel) and Alexa 488-conjugated donkey anti-rabbit IgG (green channel) antibodies. Stained cells were visualized by confocal fluorescence microscopy. The right columns show the merged images of double staining of CI-MPR and LC3. Magnification 63×. Insets show high magnification views. (B) Quantitative analysis of CI-MPR and LC3 co-localization after internalization of CI-MPR. The cells grown on coverslips were incubated at 18°C for 1 hour with 15 ng/ml Alexa 488-anti-CI-MPR monoclonal antibody. The cells were washed and fixed (0 min) or warmed to 37°C for 5, 15, 30 minutes (as indicated) before being fixed, permeabilized and stained with anti-LC3. The rate of CI-MPR/LC3 co-localization was determined using performed by LSM 3.2 software (Zeiss). The analysis was performed on at least 20 cells per sample. In severe PD fibroblasts, co-localization is higher as compared with control and CI-MPR, indicating sequestration of CI-MPR in autophagosomes.

In order to understand where the cycling of CI-MPR is arrested we investigated the nature of the peripheral structures positive for CI-MPR observed at the steady state in PD fibroblasts. We found that a significant fraction of them were positive for the autophagosome marker LC3 in intermediate and severe PD fibroblasts (Figure 6a). To gain insight into which fraction of CI-MPR came in contact with autophagosomes, whether the one moving from the TGN to the early and late endosomes, or the one recycling back from the plasma membrane, we followed this latter fraction through the internalized CI-MPR antibody. The coefficient of co-localization was analyzed in cell populations identified by the number of LC3-positive spots. In PD fibroblasts we found that over time an increasing amount of the CI-MPR antibody co-localized with LC3, particularly in the fraction of cells showing the highest accumulation of LC3-positive spots (Figure 6b). Altogether these results suggest that CI-MPR in PD fibroblasts is, at least in part, diverted towards the expanded autophagosomal or autophagolysosomal compartments.

### The uptake of rhGAA is less efficient in fibroblasts from severe and intermediate PD patients

**Figure 7:**
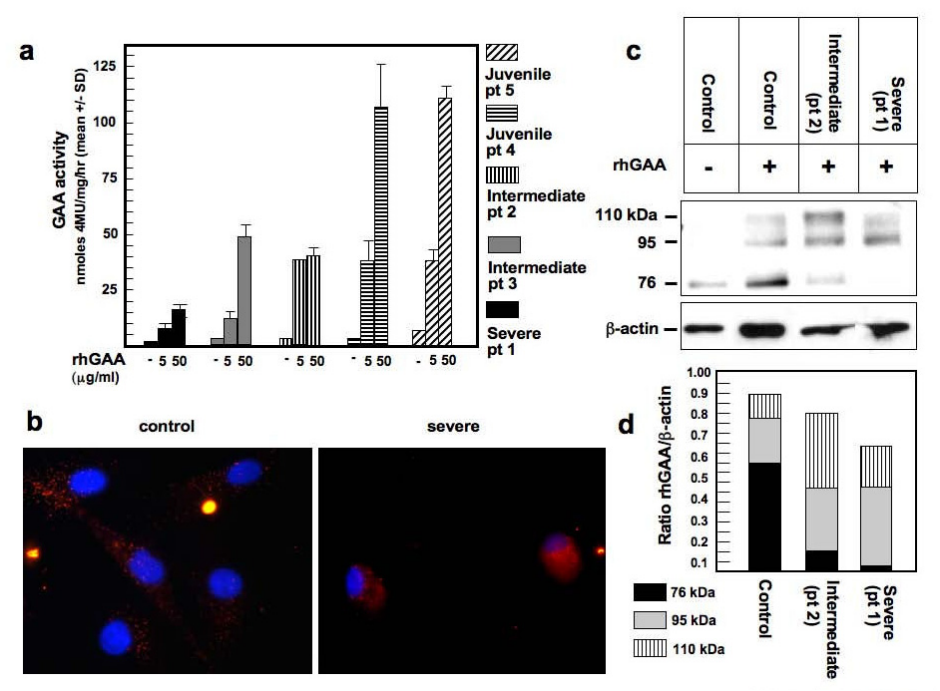
**The uptake of rhGAA is defective in fibroblasts from severe and intermediate Pompe disease patients**. (A) Pompe disease (PD) fibroblasts were incubated for 24 hours with different concentrations of rhGAA (0.5, 5, 50 μg/ml) in the medium and GAA activity was assayed. Correction of GAA activity in severe and intermediate fibroblasts from patients 1 to 3 is less efficient as compared with juvenile fibroblasts from patients 4 and 5. (B) Uptake of Alexa Fluor 546-labeled rhGAA in control and PD fibroblasts. The cells were grown on coverslips and were incubated with fluorescent rhGAA for different times (2, 4 and 8 hours), washed, fixed and visualized by fluorescence microscopy. The images show the results obtained after 2 hours of incubation. In severe PD fibroblasts a diffuse staining of labelled rhGAA is observed, in contrast with the granular pattern typical of lysosomal staining in control fibroblasts. Magnification 63×. (C) Western-blot analysis of GAA polypeptides shows reduced processing of rhGAA precursor into the 76 kDa mature enzyme, confirming impaired transport of rhGAA to the lysosomal compartment. Lysates from untreated control fibroblasts and control and PD fibroblasts treated with rhGAA for 4 hours were analyzed. (D) ImageJ quantitative analysis of the rhGAA molecular forms. The intensity of bands is normalized as ratio of rhGAA/β-actin.

As a result of a deranged CI-MPR trafficking and of a reduced availability of the receptor at the plasma membrane it could be anticipated that the uptake and targeting to lysosomes of lysosomal enzymes is impaired in PD fibroblasts. We therefore investigated the uptake of rhGAA in PD cells. Fibroblasts were cultured in the presence of variable concentrations of rhGAA for 24 hours (to obtain substantial and easily measurable enzyme activity within cells and to allow processing of the precursor into the mature active 76 kDa GAA molecular forms). Different enzyme concentrations (0.5, 5, 50 μg/ml) of rhGAA in the medium were used to study the uptake in a range of concentrations in which CI-MPR is not saturated. The correction of enzyme activity with the lowest concentration (0.5 μg/ml)  was negligible, whereas GAA activity was measurable after incubation with the highest rhGAA concentrations. Correction of GAA deficiency was less efficient in fibroblasts from patients with severe phenotypes, as compared with the activity attained in cells from juvenile patients (Figure 7a). Cells from intermediate patients showed intermediate GAA levels after incubation with rhGAA. All cell lines showed similar growth rates, suggesting that the differences in GAA uptake were not correlated to cellular growth. Uptake of rhGAA was completely abolished by the addition to the culture medium of 5 mM mannose-6-phosphate, thus confirming that enzyme uptake was CI-MPR-dependent (data not shown).

The fate of internalized GAA in different cells was also investigated by analyzing the uptake of Alexa Fluor 546-labelled rhGAA. Fibroblasts were incubated with fluorescent rhGAA for variable times (2, 4 and 8 hours), fixed and examined by fluorescence microscopy. Figure 7b shows the results obtained after 2 hours of incubation in a severe PD cell line, compared with control fibroblasts. rhGAA is taken up efficiently by control fibroblasts, where rhGAA signal appears clustered in granular structures, which probably indicates correct delivery to the lysosomal compartment. In contrast, in severe PD fibroblasts the Alexa Fluor 546 signal is dispersed and less intense. Similar results were observed in cells incubated with fluorescent rhGAA for longer periods (4 and 8 hours) (data not shown).

Evidence of an impaired targeting of rhGAA to lysosomes was also obtained by western blot analysis of rhGAA maturation in PD cells. All cells were incubated with 50 μg/ml rhGAA, harvested and cell lysates subjected to western blot analysis. The amount of the single GAA molecular forms was quantitated by ImageJ analysis. In control fibroblasts the endogenous GAA was subtracted from the total amount detected after incubation with rhGAA. In severe and intermediate PD fibroblasts the amount of the enzyme internalized was reduced, confirming that GAA uptake is less efficient as compared with control fibroblasts (Figure 7c And 7d). In addition, the processing of the enzyme was impaired in PD cells. Since the conversion of the 100 kDa to 95 kDa precursor GAA molecular forms into the mature 76 kDa enzyme takes place in the lysosomal compartment, these results indicate that the trafficking of rhGAA to lysosomes is abnormal in PD fibroblasts.

**Figure 8:**
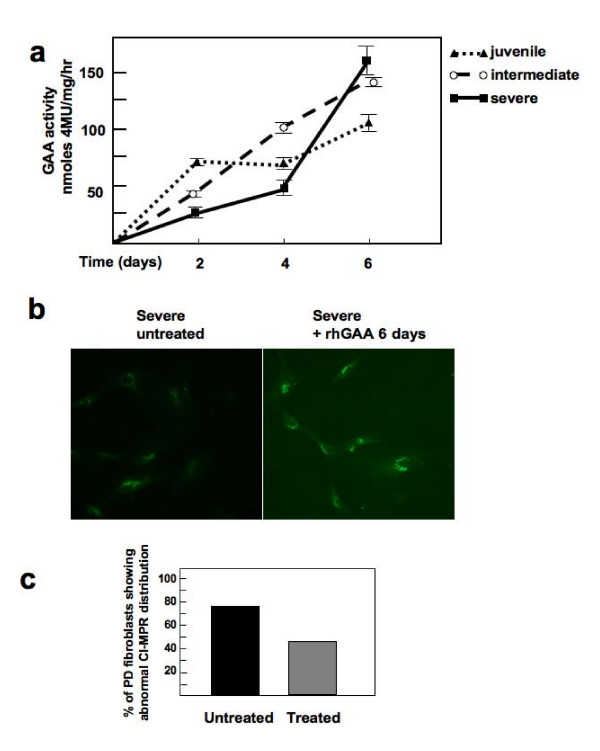
**Correction of GAA activity in Pompe disease fibroblasts**. (A) To study the time course of GAA correction, fibroblasts from Pompe disease (PD) patients were incubated in the presence of 50 μg/ml rhGAA for 2, 4 and 6 days. The medium and the enzyme were refreshed every other day. After the times indicated the cells were harvested and GAA activity measured. Untreated fibroblasts were cultured and harvested in parallel. Complete correction of GAA activity in severe PD fibroblasts requires 6 days and is slower than in the other cell lines. (B) CI-MPR intracellular distribution in untreated severe fibroblasts and severe fibroblasts treated for 6 days with rhGAA. The cells were fixed, permealized and stained with mouse monoclonal antibody to CI-MPR and Alexa 488-conjugated donkey anti-mouse IgG secondary antibody. Stained cells were visualized by fluorescence microscopy. * = *p *≤ 0.000003, chi-square test. The data were analyzed using EpiInfo (version 3.3, 2004). (C) CI-MPR distribution was scored by two independent researchers. The analysis was performed on 10 images, each containing 40 cells. After rhGAA treatment for 6 days the rate of PD cells showing a reduced and/or mislocalized signal is decreased to 40%, as compared with untreated cell (90%). *p *≤ 0.026

The studies on rhGAA uptake indicate that single rhGAA administrations are not equally efficient in correcting GAA deficiency in different PD fibroblasts, due to a derangement of the CI-MPR distribution, and contribute to the understanding of the variable response to ERT in different patients. However, it may be speculated that repeated administrations of rhGAA are more effective in correcting GAA activity and, possibly, the cellular phenotype in PD cells. We therefore studied the effects of a continuous supply of GAA in PD fibroblasts, extending the incubation time of PD fibroblasts with rhGAA for several days. To this purpose fibroblasts were incubated for 0 to 6 days and the medium containing 50 μg/ml rhGAA was refreshed every other day. Under these conditions GAA activity increased steadily and within 2 to 4 days approached normal or near-normal values in intermediate and juvenile PD cell lines. In cells from a patient with severe PD a complete correction of GAA activity required a longer incubation with rhGAA, and GAA activity showed a 'catch-up' increase between days 4 and 6, eventually reaching normal values (Figure 8a). This suggests severely affected PD cells may be more difficult to treat and may require additional duration of therapy.

In the severe PD cell line we also studied whether prolonged incubation with rhGAA is effective in rescuing the abnormal CI-MPR distribution. To this purpose we performed an immunofluorescence analysis of CI-MPR in these cells after 6 days of rhGAA treatment (see above). Scoring of CI-MPR distribution showed that the number of cells showing a reduced and/or mislocalized signal had decreased after treatment to approximately 44%, while in untreated cells cultured in parallel the rate of cells showing abnormal CI-MPR localization had remained unchanged (approximately 73%) (Figure 8b). This indicates that ERT is effective in rescuing the cellular phenotype and that a normalization of the CI-MPR distribution is essential for correction of GAA activity. The rapid 'catch-up' increase of GAA activity observed in the severe cell line between 4 and 6 days is probably the consequence of a restored CI-MPR distribution and function.

### The uptake of rhGAA is less efficient in fibroblasts from other LSDs

It is possible to speculate that a derangement of cellular functions and morphology, triggered by intralysosomal substrate storage, is a generalized phenomenon in LSDs. This issue is important for possible implications in the diseases for which ERT is available. Although our work was focused on a single model of LSD, PD, we also studied the uptake of rhGAA in cells from other LSDs.

**Figure 9:**
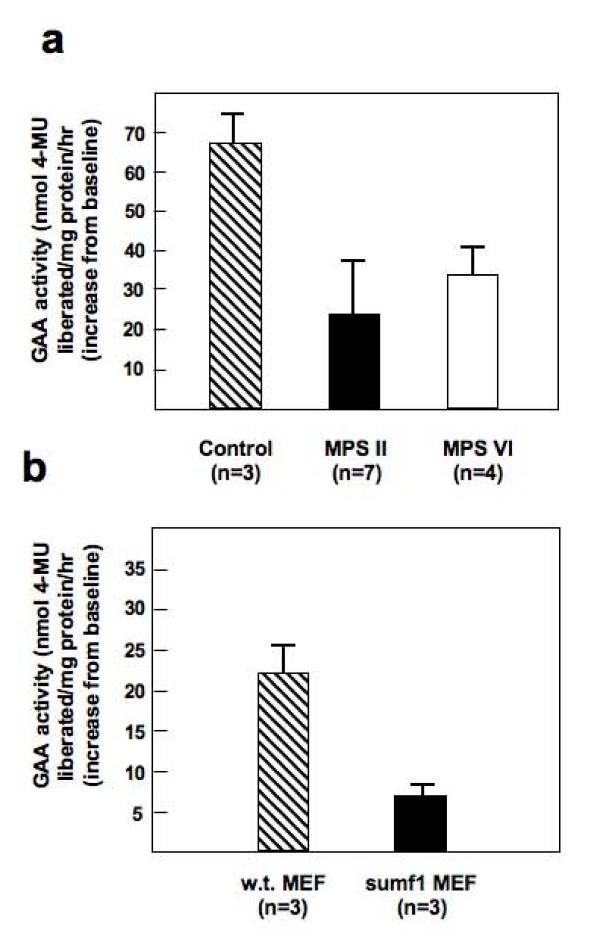
**The uptake of rhGAA is defective in fibroblasts from other lysosomal storage disorders**. (A) Human control fibroblasts and fibroblasts from MPSII and MPSVI patients and (B) mouse embryonic fibroblasts (MEF) from control mice and *Sumf1*^-^/^- ^mice were incubated with rhGAA (50 μg/ml) for 24 hours. GAA activity is expressed as nmol 4-methylumbelliferone liberated/mg protein/h. Activities are expressed as the difference between the activity after 24 hours of incubation and baseline activity. All mutant cell lines analyzed show reduced GAA activity as compared with control cells.

We studied rhGAA uptake in fibroblasts from two mucopolysaccharidoses (MPS), Hunter disease (MPS II) and Maroteaux-Lamy disease (MPS VI), both characterized by generalized accumulation of glycosaminoglycans, and in mouse embryonic fibroblasts (MEFs) from a mouse model of multiple sulphatase deficiency (MSD), obtained by disruption of *Sumf1 *[25]. In all of these cell lines rhGAA, expressed as the difference between baseline activity and the activity after incubation with the recombinant enzyme for 24 hours, appeared reduced as compared with that observed in control fibroblasts (Figure 9a) and in control MEFs (Figure 9b).

## Discussion

We have demonstrated that fibroblasts from patients with PD show abnormalities of cellular morphology, including intracellular storage of glycogen, expansion of the autophagic compartment, disorganization of the Golgi apparatus and of the TGN, and disruption of intracellular CI-MPR localization. The derangement of CI-MPR distribution apparently correlates with disease severity and has functional consequences, resulting in an impaired uptake of exogenous rhGAA in cells from early-onset PD patients. The demonstration *in vitro *of a variable efficacy of rhGAA in correcting GAA activity in different cell lines has implications for the efficacy of ERT *in vivo*.

Although cellular pathology in PD has been mostly investigated in muscle cells, the observation of intracellular storage in cultured PD fibroblasts is not unexpected. It has been shown that fibroblasts, an *in vitro *system that can be obtained by procedures that cause only mild discomfort to patients, do accumulate glycogen and that GAA deficiency and glycogen storage correlates with age at onset and clinical course of the disease [26]. In PD fibroblasts we observed glycogen storage not only in lysosomes and in MVBs, but also in the cytoplasm. This may be a consequence of a reduced clearance of cytoplasmic glycogen by a less efficient autophagic machinery.

The autophagic compartment was in fact abnormal in PD fibroblasts, particularly in cells from severe and intermediate patients. Autophagy is a physiological catabolic pathway through which cytosolic proteins and organelles, such as mitochondria, are sequestered by double-membrane vesicles (autophagosomes or autophagic vesicles) and degraded in the lysosomal compartment after autophagosome-lysosome fusion [27]. An impairment of autophagy has recently been implicated in the pathophysiology of LSDs, with mechanisms that remain still partially understood [28-30].

In PD, an age-dependent expansion of the autophagosomal compartment was observed in a PD knock-out mouse model obtained by disruption of the murine *Gaa *gene and in muscle biopsies from PD patients [31-34]. These studies showed for the first time the presence of abnormal autophagy in PD tissues and correlated the presence of such abnormalities with a poor response to ERT, although the abnormalities of the autophagosomal compartment appeared to be confined to type II muscle cells. Our results add further information on the derangement of the autophagic pathway in PD as they indicate that abnormalities of autophagy are a generalized phenomenon in PD, detectable in cells other than specific muscle cells and that fibroblasts may represent a useful tool for *in vitro *studies aimed at investigating the mechanisms underlying these abnormalities.

We found abnormalities of Golgi and TGN morphology both by EM analysis and by immunofluorescence studies using known markers of these organelles. A large number of genes are involved in the correct organization of the Golgi apparatus [35,36] and it might be an intriguing and challenging task to identify putative targets of glycogen storage among these genes and thus clarify the mechanisms leading to Golgi abnormalities in PD. In principle, an abnormal retrograde trafficking of membranes may play a role in disrupting the normal organization of the Golgi apparatus, as it has been shown that this mechanism is important in allowing the turnover of membranes and membrane-bound proteins and in maintaining the integrity of cellular organelles [37].

The abnormalities of Golgi apparatus and the TGN may explain, at least in part, the changes in the distribution of CI-MPR, a membrane-bound protein mostly localized in Golgi and the TGN. CI-MPR traffics within cells following different itineraries in which transit to the TGN is an important stage, such as the lysosomal enzyme biosynthetic pathway, from the TGN to the late endosomes and back to the TGN; the endosomal pathway from the plasma membrane to the ERC, where most of it (84%, [38]) recycles back to the plasma membrane; and an additional route that diverges from the endocytic pathway and reaches late endosomes and from these organelles carries CI-MPR again to the TGN [39].

The abnormal CI-MPR distribution observed in PD fibroblasts may be the result of a derangement in one or more of these pathways. We focused on the endocytic pathway, as this route is important for ERT. The rationale of ERT relies upon the demonstration that exogenous lysosomal enzymes are internalized through the interaction of mannose or mannose-6-phosphate ligands, present on the carbohydrate chains of the enzymes, with their specific receptors. An intact and efficient mannose-6-phosphate pathway is therefore essential for the efficacy of such therapeutic approach and it could reasonably be anticipated that an impaired trafficking of CI-MPR through the endocytic pathway may impact the efficiency of lysosomal enzyme uptake and thus the efficacy of ERT *in vivo*.

Time-lapse experiments using a severe PD cell line, showing the most striking abnormalities of cellular phenotype and of CI-MPR intracellular distribution, indicated that the route from the plasma membrane to the TGN is impaired. This may be the consequence of an abnormal retrograde trafficking of the CI-MPR from the late endosomes to the TGN. CI-MPR traffics together with Tf and TR, in the early endosomal compartment and in the ERC, where they recycle back to the plasma membrane. Since we did not find abnormalities of Tf and TR distribution in the PD cells examined, it is likely that the disruption of CI-MPR trafficking occurs at the late endosomal compartment and in the retrograde route to the TGN. In this respect it is worth mentioning that abnormal retrograde CI-MPR transport (such as due to inhibition of PACS1 and AP1), resulted in disruption of its normal localization [40-42].

**Figure 10:**
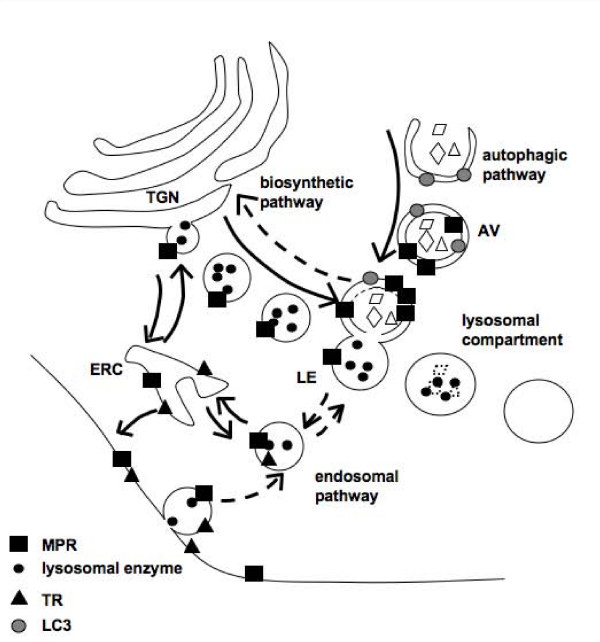
**Schematic representation of CI-MPR trafficking in the cells**. CI-MPR follows different routes, including the biosynthetic pathway from the trans-Golgi network (TGN), where it binds and delivers newly synthesized lysosomal hydrolases to the late endosomal compartment. A fraction (approximately 10%) of CI-MPR is located on the cell surface, where it mediates the uptake of lysosomal enzymes, is internalized in the endocytic pathway and recycles through the endocytic recycling compartment (ERC), again to the plasma membrane. The finding of increased co-localization of CI-MPR with LC3 in Pompe disease (PD) fibroblasts indicates that in these cells part of the CI-MPR is diverted from its normal routes and is sequestered in autophagosomes and autophagolysosomes. Since transferrin and transferring receptor trafficking is not affected in PD fibroblasts, it is likely that the disruption of CI-MPR trafficking occurs at the late endosomal compartment and in the retrograde route to the TGN.

The finding of increased co-localization of CI-MPR with LC3, a marker of the autophagosomes and autophagolysosomes, supports the hypothesis of an impaired retrograde trafficking of CI-MPR from late endosomes to the TGN, and indicates that, at least in part, CI-MPR is sequestered in relatively inert vesicles deriving from the fusion of autophagosomes with substrate-engulfed lysosomes. Sequestration of CI-MPR in these organelles leads to a depletion of functionally available CI-MPR at the plasma membrane (Figure 10).

A consequence of the reduced availability of CI-MPR at the plasma membrane is that the uptake of exogenous enzymes is less efficient. Defective uptake of another lysosomal hydrolase, acid sphingomyelinase, was demonstrated by Dhami and Schuchman in macrophages from a Niemann-Pick A mouse model [43]. The authors hypothesized that lipid accumulation in macrophages leads to abnormalities in CI-MPR trafficking and/or degradation, although they did not provide a morphological analysis of CI-MPR distribution and trafficking in their cell system supporting this hypothesis.

We showed that correction of GAA activity by rhGAA was impaired in individual PD cell lines. In principle, poor correction of GAA activity may result from impaired uptake or from reduced enzyme maturation and stability. The combination of the results of uptake studies of fluorescent-labelled enzyme and the results of western blot analysis of rhGAA processing indicates that both mechanisms contribute to a reduced efficacy of ERT in PD.

In our studies the rate of rhGAA uptake by PD fibroblasts varied among different cell lines and correlated with the degree of abnormality of CI-MPR distribution. We suggested that the abnormalities of CI-MPR distribution and GAA uptake correlate with disease severity, although other factors may also be implicated. The observation that cells from PD patients with the severe phenotypes are less efficient in internalizing rhGAA is apparently in contrast with the observation that the best results with ERT are obtained in patients with the infantile classic PD. In fact, in these patients treatment is started at an earlier age as compared with late-onset patients, and it is reasonable to think that if the abnormalities of cell functions are triggered by substrate storage, then the start of treatment before extensive storage occurs, particularly in the tissues which are the main targets of disease (cardiac and skeletal muscle), may prevent the development of these abnormalities.

The implications of our results for treatment with ERT in PD are evident. ERT has proved to be particularly challenging in a subset of PD patients, whereas in other patients the efficacy of ERT was excellent. The reasons for such a variable response to ERT are still controversial. Age at start of treatment is surely one of the most important factors for the outcome of ERT. In addition, other factors have been proposed. An effect due to the poor response of type II muscle fibres, initially suggested by Fukuda et al [31] was not confirmed by a subsequent study [44]. The role of other factors such as different genetic backgrounds and the different impacts of *GAA *gene mutations has not been fully investigated.

Our results identify the role of additional factors, such as the derangement of cellular functions and disruption of CI-MPR localization, implicated in the variable response to ERT.

ERT is based on weekly or biweekly infusions, depending on treatment protocols specific for each LSD. In PD, clinically detectable results are expected to be evident only after long-term treatments. An important question is whether correction of GAA deficiency by ERT may start a virtuous mechanism by correcting cellular abnormalities, restoring normal function of the CI-MPR pathway and, in turn, further improving the efficacy of rhGAA uptake. To address this question we incubated PD fibroblasts with rhGAA for long periods, up to 6 days. This resulted in increased GAA activity in all cell lines. However, correction was hampered in the cells with prominent abnormalities of CI-MPR distribution. In these cells a 'catch-up' increase, eventually leading to normal intracellular GAA activity, was observed in association with an improved CI-MPR cellular distribution.

This experiment provides useful information about ERT. First, it further supports the correlations between CI-MPR distribution and efficacy of ERT. In addition, our results support the idea that ERT may correct the abnormalities of cellular morphology and function. On the other hand, it should be mentioned that the experimental conditions we used, with relatively high rhGAA concentrations in the culture medium and continuous supply of new enzyme, differ from those used in human therapy. PD patients (as well as patients with other LSDs treatable with ERT) are treated with single rhGAA infusions every week or every other week and the concentrations attained in plasma return to baseline levels a few days after the infusion. Our results may therefore suggest that ERT treatment protocols should be reconsidered and that other approaches, such as gene therapy (providing a continuous supply of enzyme either locally or from 'factory' organs) may result in more effective rescuing of cell and tissue damage.

An intriguing question is whether the results obtained in PD fibroblasts indicate a mechanism shared by other LSDs, or are the result of the storage of a specific substrate. Although it was not in the scope of this study to provide a general model for all LSDs, we investigated whether rhGAA uptake is also affected in fibroblasts from patients with other LSDs. To this purpose we studied the uptake of rhGAA by MEFs from an animal model of MSD, generated by the disruption of the *Sumf1 *gene [25]. This model seems particularly suitable to address this point as in MSD the simultaneous deficiency of an entire family of lysosomal hydrolases causes substantial storage of a range of sulphated substrates. Also in these cells, and in fibroblasts from MPS II and VI, the uptake of rhGAA was less efficient as compared with controls. These observations are in favour of the hypothesis of a common abnormality in LSDs and indicate the need for similar studies, with the specific recombinant enzymes, in cell lines and animal models of other LSDs.

## Conclusion

Recent studies have shown that in LSDs substrate storage triggers secondary events leading to abnormalities of cell morphology and function. In fibroblasts from PD we demonstrated that a consequence of these abnormalities is a deranged distribution and function of the CI-MPR. These *in vitro *data may have implications for the response to ERT in individual patients and should be investigated *in vivo *in PD patients and in other LSDs. Our studies may also indicate the need for therapies targeted to these cellular abnormalities. If a strategy to improve cellular functions is found, this may have a significant impact on the efficacy of ERT and may suggest a multi-drug therapeutic approach tailored to individual patients.

## Methods

### Cell lines

Cells from a classic infantile (patient 1; [45]), two intermediate (patients 2 and 3; 1 and 2 in [46], respectively) and two juvenile (patients 4 and 5; cases 3 and 5 in [45], respectively) PD patients were available in the laboratory of the Department of Paediatrics, University of Naples, Italy. Each experiment was performed in all cell lines, although for some of the experiments data from single patients of each phenotype are shown. The experiments on CI-MPR, Tf and TR trafficking and on CI-MPR immunolocalization after long-term rhGAA correction were only done in the cell line from patient 1, which showed the most striking abnormalities of cell morphology.

Fibroblasts from patients with MPS II and MPS VI were provided by M Filocamo, G Gaslini Institute and Telethon Genetic Biobank Network, Genoa, Italy (Telethon grant GTB07001A). *Sumf1*^-/- ^MEFs, and control MEFs were available at the Telethon Institute of Genetics and Medicine, Naples, Italy. Normal age-matched control fibroblasts, available in the laboratory of the Department of Paediatrics, University of Naples, Italy, were studied for comparison.

All cell lines were grown at 37°C with 5% CO_2_, in Dulbecco's modified Eagle's medium (DMEM, from Invitrogen, NY, USA) and 10% foetal bovine serum (FBS) (Sigma-Aldrich, St Louis, MO, USA), supplemented with 100 U/ml penicillin and 100 μg/ml streptomycin. To induce starvation and stimulate autophagy, fibroblasts were cultured under amino acid deprivation in Earle' Balanced Salt Solution (from Invitrogen, NY, USA) for 2 hours, before being analyzed.

### Reagents

The primary antibodies used for immunofluorescence and western blot analysis were: anti-LC3 rabbit polyclonal antiserum (Novus Biologicals, Littleton, CO, USA); anti-β-actin mouse monoclonal antibody (Sigma-Aldrich); anti-human LAMP2 mouse monoclonal antibody (Santa Cruz Biotechnology, CA, USA); anti-human CI-MPR mouse monoclonal antibody, anti-human TGN38 goat polyclonal antiserum and anti-human mannosidase II rabbit polyclonal antiserum (Novus Biologicals); anti-GM130 mouse monoclonal antibody (BD Transduction Laboratories); anti-golgin-97 rabbit polyclonal antiserum (provided by Edward KL Chan); anti-TGN46 sheep polyclonal antiserum (Serotec); anti-γ-adaptin mouse monoclonal antibody (BD Transduction Laboratories); anti-transferrin receptor mouse monoclonal antiserum (Zymed Laboratories). Secondary antibodies were: anti-rabbit, anti-mouse and anti-goat conjugated to Alexa Fluor 488 or 596 (Molecular Probes, Eugene, OR, USA); HRP-conjugated anti-rabbit or anti-mouse IgG (Amersham, Freiburg, Germany).

Labelling of rhGAA and anti-CI-MPR antibody was performed by using a Protein Labelling Kit and Alexa Fluor 488 Monoclonal Antibody labelling kit (Molecular Probes, Eugene, OR, USA), respectively. rhGAA (Myozyme) was purchased from Genzyme Co (Naarden, the Netherlands).

### Immunofluorescence studies

To study the distribution of LC3, CI-MPR, cis-Golgi and TGN markers, Tf and TR, human fibroblasts grown on coverslips were fixed using methanol, permeabilized using 0.1% saponin and blocked with 0.01% saponin, 1% FBS diluted in PBS for 1 hour. The cells were incubated with the primary antibodies, with secondary antibodies in blocking solution and then mounted with vectashield mounting medium with DAPI (Vector Laboratories, Burlingame, CA).

For experiments shown in Figures 4 and 6 photographs were taken by using a Zeiss Axioplan 2 fluorescence microscope (Carl Zeiss, Jena, Germany) integrated with the AxioCam MR camera and 63× oil objective with a 1.25 numerical aperture lens. Digital images were captured by using a digital colour Zeiss Axiocam 1300×1030 pixel and Zeiss AxioVision software. For experiments shown in Figures 3 and 5 confocal microscopy analysis was performed using a Leica laser-scanning confocal image system TCS SP2 AOBS (Leica Microsystems, Heidelberg, Germany). Samples were excited with a 488 nm Ar laser and a 594 nm He-Ne laser. Samples were vertically scanned from the bottom coverslip with a total depth of 50 μm and a 63× (1.32 NA) HP PLAPO oil-immersion objective. A total of 10 z-line scans with a step distance of 0.2 mm was collected and maximum intensity projections were generated with Leica Confocal Software (Leica Microsystems, Heidelberg, Germany).

In experiments shown in Figures 4 and 8 CI-MPR distribution was scored by two independent researchers. Results of these analyses were obtained on a total of 40 cells for each cell line.

### Electron microscopy analysis

Cells were fixed for 30 minutes (room temperature) with 1% glutaraldehyde prepared in Hepes 0.2 M and processed for EM as described previously [47]. The percentage of endolysosome volume occupied by glycogen was measured using AnalySIS software (Soft Imaging Systems GmbH, Munster, Germany).

### Western blot analysis

To study GAA processing and LC3 maturation, fibroblast extracts were subjected to western blot analysis. The cells were harvested, washed in PBS and lysed in cold RIPA buffer containing Complete Protease Inhibitor Cocktail (Roche Diagnostics, Mannheim, Germany) for 30 minutes on ice. Lysates were precleared by centrifugation at 10,600 g for 10 minutes at 4°C. Equal amounts (20 μg protein) of fibroblast extracts were subjected to SDS-PAGE electrophoresis (7% or 10% acrylamide in different experiments) and proteins were transferred to PVD membrane (Millipore, Billerica, MA, USA). Anti-human GAA and anti-human LC3 rabbit polyclonal antisera were used as primary antibodies to detect GAA polypeptides and LC3-I and LC3-II (Novus Biological, Littleton, CO, USA), respectively; to detect β-actin a monoclonal mouse antibody was used. Immunoreactive proteins were detected by chemiluminescence (ECL, Amersham, Freiburg, Germany). Western blots show representative results from one of three experiments. Quantitative analysis of band intensity was performed using ImageJ.

### FACS analysis

To obtain a quantitative analysis of CI-MPR availability at the plasma membrane, PD and control fibroblasts were suspended in blocking buffer (1% FBS in PBS). After incubating for 30 minutes on ice, the cells were washed and suspended in blocking buffer containing titered excess of CI-MPR monoclonal antibody for 30 minutes on ice. Cells were then washed, re-suspended in blocking buffer containing phycoerythrin-conjugated anti-mouse secondary antibody for 30 minutes on ice, filtered through 70 μm filcons (BD Biosciences Pharmingen, San Jose, CA, USA) and analyzed using a BD Biosciences FACS Aria (BD Biosciences Pharmingen, San Jose, CA, USA).

### Uptake of rhGAA and correction of GAA activity

To study rhGAA uptake and correction of GAA activity PD, MPS II and MPS VI fibroblasts were incubated with different concentrations (0.5, 5, 50 μg/ml) of rhGAA for 24 hours. The cells were then harvested and cell pellets were washed twice with PBS, resuspended in the water and disrupted by five cycles of freeze-thawing. GAA activity was assayed as already described [45]. Protein concentrations were determined in total homogenates by the Bradford assay (Bio-Rad, Hercules, CA).

To study the time course of GAA correction, fibroblasts from a patient with severe PD were incubated in the presence of 50 μg/ml rhGAA for 2, 4 and 6 days. The medium and the enzyme were refreshed every other day. After the times indicated the cells were harvested and GAA activity assayed as indicated above. Untreated cells were cultured and analyzed in parallel. Part of the cells was analyzed for CI-MPR localization as indicated above.

To study the uptake of fluorescent rhGAA, the enzyme was labelled and purified using the Alexa Fluor 546 Protein labelling kit (Molecular Probes), according to the manufacturer's instructions. Fibroblasts were incubated with rhGAA (50 μg/ml) for different times 2, 4 and 8 hours. The cells were fixed in methanol for 5 minutes and examined on a fluorescence microscope Axioplan 2 (Zeiss, Thornwood, NY, USA) as described above.

### Endocytic recycling of CI-MPR and transferrin uptake

To study trafficking through the endocytic pathway, fibroblasts were incubated in DMEM 20 mM Hepes without FCS with 15 ng/ml Alexa 488-anti-CI-MPR antibody for 60 minutes at 18°C. The cells were washed in DMEM 20 mM Hepes with 10% FCS and fixed (0 min) or warmed to 37°C for 5, 15, 30, 60 minutes before fixation. The cells were permeabilized and stained with Alexa Fluor 488 anti-mouse IgG to amplify the signal or with anti-LC3 primary antibody and Alexa Fluor 594 anti-rabbit IgG. Stained cells were visualized by confocal fluorescence microscopy as indicated. Quantitative analysis of CI-MPR fluorescence intensity of was performed by LSM 3.2 software (Zeiss). The percentage of CI-MPR/LC3 co-localization was determined using by LSM 3.2 software (Zeiss). The analysis was performed on 40 cells.

To study the uptake of Tf, cells were incubated with 50 μg/ml Alexa 488-transferrin for 60 minutes at 18°C, as indicated, fixed and stained with TR antibody and Alexa Fluor 594 anti-rabbit IgG.

## Competing interests

The authors declare that they have no competing interests.

## Authors' contributions

MC performed the immunofluorescence and western blot studies, CI-MPR trafficking and contributed to the design of the study and the interpretation of results. CP performed the experiments on rhGAA uptake and on correction of GAA activity in PD cells. AT contributed to the immunofluorescence studies and the statistical analysis of the results. MV contributed to the CI-MPR and transferrin trafficking studies. BR performed cell culturing and contributed to western blot analysis. EP performed the electron microscopy analysis. FD performed rhGAA uptake studies in cells from other LSDs. GA contributed to the design of the study and the interpretation of data. MADM contributed to the design of the study, interpretation of data and revision of the manuscript. GP conceived the study and participated in its design, interpretation of results and drafting of the manuscript. All authors have read and approved the final manuscript.
